# MRI radiomic features of peritumoral edema may predict the recurrence sites of glioblastoma multiforme

**DOI:** 10.3389/fonc.2022.1042498

**Published:** 2023-01-04

**Authors:** Hao Long, Ping Zhang, Yuewei Bi, Chen Yang, Manfeng Wu, Dian He, Shaozhuo Huang, Kaijun Yang, Songtao Qi, Jun Wang

**Affiliations:** ^1^Department of Neurosurgery, Nanfang Hospital, Southern Medical University, Guangzhou, China; ^2^The First Clinical Medicine College, Southern Medical University, Guangzhou, China; ^3^Department of oncology, Guangdong 999 Brain Hospital, Guangzhou, China; ^4^Neural Networks Surgery Team, Southern Medical University, Guangzhou, China

**Keywords:** glioblastoma, recurrence, radiomics, peritumoral edema, heterogeneity

## Abstract

**Background and purpose:**

As one of the most aggressive malignant tumor in the central nervous system, the main cause of poor outcome of glioblastoma (GBM) is recurrence, a non-invasive method which can predict the area of recurrence pre-operation is necessary.To investigate whether there is radiological heterogeneity within peritumoral edema and identify the reproducible radiomic features predictive of the sites of recurrence of glioblastoma(GBM), which may be of value to optimize patients’ management.

**Materials and methods:**

The clinical information and MR images (contrast-enhanced T1 weighted and FLAIR sequences) of 22 patients who have been histologically proven glioblastoma, were retrospectively evaluated. Kaplan-Meier methods was used for survival analysis. Oedematous regions were manually segmented by an expert into recurrence region, non-recurrence region. A set of 94 radiomic features were obtained from each region using the function of analyzing MR image of 3D slicer. Paired t test was performed to identify the features existing significant difference. Subsequently, the data of two patients from TCGA database was used to evaluate whether these features have clinical value.

**Results:**

Ten features with significant differences between the recurrence and non-recurrence subregions were identified and verified on two individual patients from the TCGA database with pathologically confirmed diagnosis of GBM.

**Conclusions:**

Our results suggested that heterogeneity does exist in peritumoral edema, indicating that the radiomic features of peritumoral edema from routine MR images can be utilized to predict the sites of GBM recurrence. Our findings may further guide the surgical treatment strategy for GBM.

## Highlights

Peritumoral edema has heterogeneity.
The heterogeneity of peritumoral edema is related to the recurrence of glioblastoma.
Radiomics may predict the potential site of glioblastoma recurrence.

## Introduction

Glioblastoma (GBM) is the most aggressive malignant tumor in the central nervous system. In order to avoid recurrence after surgery, maximal safe resection is always performed, the effect of which is affirmative. A recent study indicated that wider surgical resection was associated with better prognosis (1). Additionally, a meta-analysis suggested that gross total resection was superior to subtotal resection in improving the patients’ prognosis (2). However, excessive resection may lead to complications. Excessive removal of functional brain areas will significantly decrease the patients’ quality of life. Accordingly, it is necessary to find a new method to optimize surgical resection.

Notably, recurrences, as the main cause of poor outcome despite the complex surgical resection and adjuvant radiochemotherapy (3), mainly occur in the area of peritumoral edema (4). Thus, in order to improve the prognosis of this aggressive tumor, it is essential to optimize surgical resection and remove the peritumoral edema area with high risk of recurrence. In reality, GBM heterogeneity, which has been the hot topic in neuro-oncology lately, is not limited to the intratumoral region but also involves the peritumoral edema (5), and this heterogeneity can be used to identify the potential recurrence region. Many studies have investigated the cellular and molecular characteristics of the peritumoral edema *via* genomic, transcriptomic, or proteomic analyses (6–8). However, considering the low sensitivity, histopathological examination is not appropriate for identifying potential recurrence regions. Furthermore, the characteristics of a sample cannot properly represent the peritumoral edema as a whole.

Radiomics, a recent method that aims at extracting features from medical images using data-characterization algorithms, not only has the potential to reflect the underlying characteristics of peritumoral edema, but is also easier to perform (9). Compared with traditional radiographic images, digital images can be converted into quantitative radiomic image features *via* high-throughput computing. These radiomic features can partially reflect the biological features and underlying pathophysiology of the relevant tissues. After decades of development, radiomics has been applied in predicting the treatment response and outcomes of cancer, tumor staging, identifying tissue, and assessment of cancer genetics (10), including GBM (11) and grade II and III gliomas (12). Radiomics can also be used for tumor regional heterogeneity analysis (13). However, the association of GBM recurrence and certain radiomic features has rarely been investigated. Moreover, it has been shown that preoperative MRI parameters contribute to prediction of the prognosis of patients with GBM (14).

Therefore, this study aimed to identify heterogeneity within the peritumoral edema region *via* radiomics, which may be beneficial to the optimization of surgical resection.

## Materials and methods

### Study population

Ethics committee approval and patient consent were obtained. We retrospectively reviewed the data of patients diagnosed with GBM from April 2008 to October 2016 in our department. Patients were included if they met all of the following criteria: (1) histopathologically confirmed diagnosis of GBM based on the World Health Organization histological grading system by at least one expert pathologist; (2) available preoperative images before the initial surgery, including contrast-enhanced T1-weighted and three-dimensional (3D) fluid-attenuated inversion recovery (FLAIR) images; (3) no residual tumor detected after the operation; (4)pathologically and image confirmed tumor recurrence,and (5) available images of tumor recurrence before reoperation (including contrast-enhanced T1-weighted and FLAIR images). The follow-up data of two patients from The Cancer Genome Atlas (TCGA) database were used to test our hypothesis.

### Endpoint

We considered OS and progression-free survival (PFS) as the endpoints of our study. OS was defined as time from treatment start to death or time from treatment start to the end of our study. PFS was defined as the time from treatment start to progression or death or time from treatment start to the end of our study, whichever occurred first.

### MRI acquisition and segmentation

All MR images of all eligible patients were obtained using the same MRI scanner (Discovery ST, GE Healthcare) according to the standard clinical scanning protocols at our hospital. Two types of MRI sequences, (1) contrast-enhanced T1-weighted spin-echo image (T1-CE): repetition time/echo time (TR/TE), 2283.9/24.3 msec confirming GBM recurrence; (2)FLAIR: repetition time/echo time (TR/TE), 7502.0/122.1 msec indicating peritumoral edema regions pre-operation, were selected for the study.

**Figure 1:**
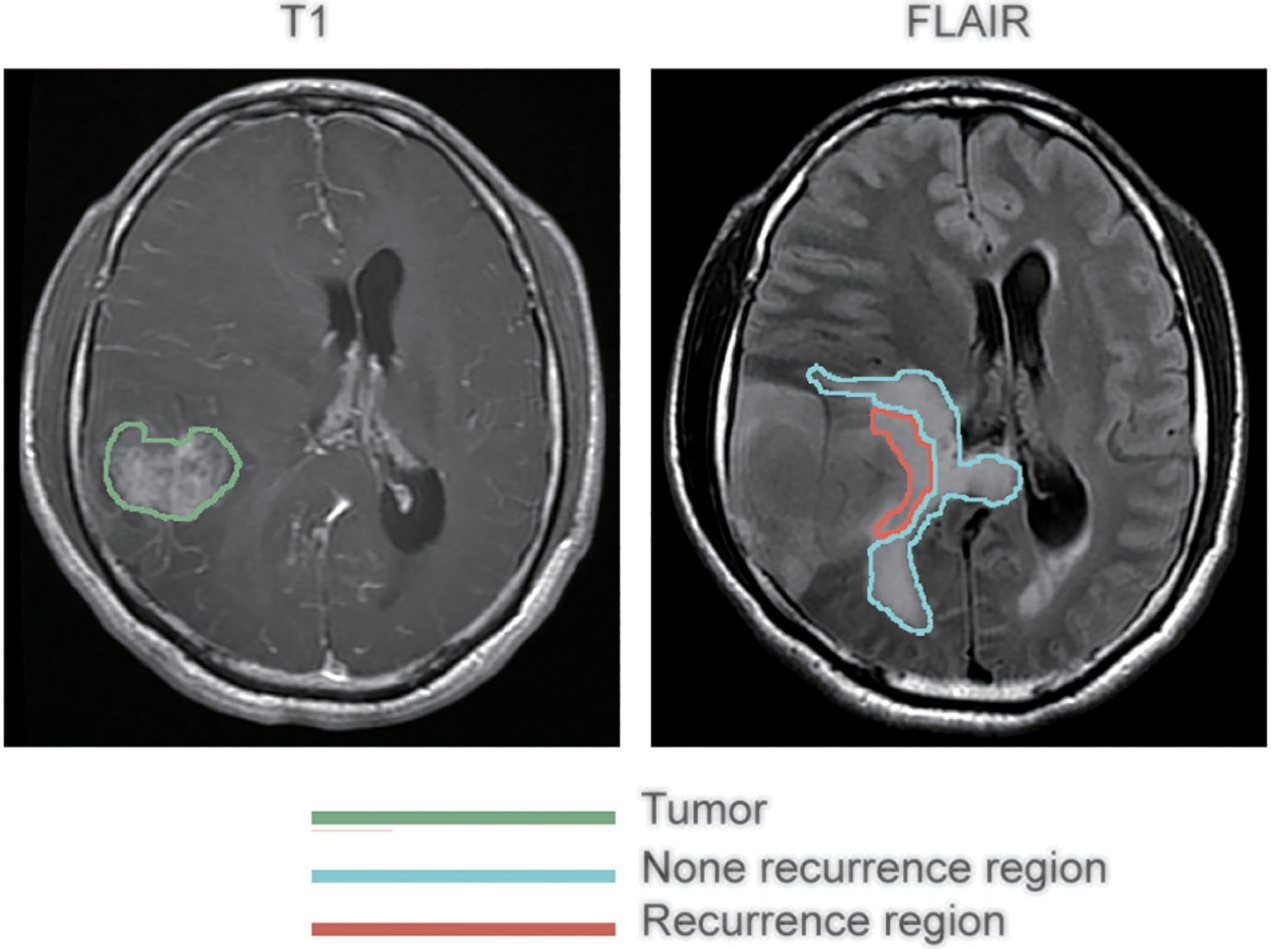
Segmentation of peritumoral edema. The annotations for tumors as delineated on contrast-enhanced T1 weighted images are outlined in green, while the annotations for recurrence and non-recurrence regions within the peritumoral edema as delineated on fluid-attenuated inversion recovery images are outlined in blue and red, respectively.

Preprocessing of the images was conducted before analysis to reduce bias. The regions of peritumoral edema were delineated on the FLAIR images. The lesions of recurrence were delineated on contrast-enhanced T1-weighted images. All these were conducted by an experienced neurosurgeon using 3D Slicer 5.2.0 (http://www.slicer.org) operating on Mac OS X. ‘Registration’ function of 3D Slicer was conducted firstly to eliminate different sequences shift induced by the movement of the patient’s head during MR scanning. That way, the two MRI images, contrast-enhanced T1-weighted and FLAIR, of each patient were guaranteed to locate on the same slicer. Subsequently, with reference to the location of the recurrent tumor on contrast-enhanced T1-weighted images, the peritumoral edema regions were divided into two subregions on the FLAIR images: recurrence and non-recurrence regions. **Figure 1** shows representative contrast-enhanced T1-weighted and FLAIR images with annotations for the tumor and the recurrence and non-recurrence regions. The area in the green coil represents the primary tumor in T1-CE image, the area in the red and blue coil represents the whole peritumoral edema regions before first operation, the area in the red coil represents the location tumor recurrence in peritumoral edema while the area in the blue coil represents other peritumoral edema regions in FLAIR sequences.

### Radiomic feature extraction and selection

Quantitative radiomic features were extracted from the recurrence and non-recurrence regions on the FLAIR images by 3D Slicer using the “Slicer Radiomics” plugin. 94 texture features that could be divided into five groups were extracted from MR images. Group 1 (shape) consisted of 16 descriptors that reflect the morphological characteristics of tumor and recurrence and non-recurrence regions. Group 2 (first order) consisted of 19 descriptors that describe the distribution of voxel intensities by frequently-used basic metrics within the MR image. Group 3 (GLCM) consisted of 26 descriptors. Group 4 (GLRLM) consisted of 16 descriptors. Group 5 (GLSZM) consisted of 16 descriptors. The descriptors in group 3,group 4 and group 5 were calculated by specific matrix algorithm.

To standardize the feature value, we converted it into standardized value according to the formula |a−b|/a×100%, where “a” stands for the value of the entire peritumoral edema region, while in analysis radiomics features from normal brain region, ‘‘a’’ represents the feature values extracted from the entire normal brain region, and “b” stands for the value of the subregions (recurrence or non-recurrence). Subsequently, the standardized values were used to identify the features with a significant difference between the recurrence and non-recurrence regions using the paired t test.

### Statistical analysis

Survival analysis was performed using Kaplan-Meier’s method. Radiomics features were examined whether followed a gaussian distribution by Shapiro-Wilk test. Then, the radiomic characteristics of the two groups were compared using paired t test. The differences were considered significant at p < 0.05. All analyses were performed using IBM SPSS Statistics v23.0 (IBM Corp.).

## Results

### Clinicopathological characteristics

A total of 22 patients with recurrent GBM were included in this study,among which 19 (86%) was male while 3 (14%) were female. The median age at diagnosis was 49 (range, 23–67).

Regarding the tumor location, 3 (14%) patients had tumors in the parietal lobe, 6 (27%) in the frontal lobe, 7 (32%) in the temporal lobe, 2 (9%) in the occipital lobe, and 3 (14%) in other regions. The Ki-67 proliferation index was lower than 50% in 13 (59%) patients, whereas 2 (9%) patients had no available data. The O6-methylguanine-DNA methyltransferase and IDH1 status was positive in 11 (50%) and 12 (55%) patients, and negative in 10 (46%) and 9 (41%) patients, respectively (**Table 1**).

**Table 1:** The clinical characteristics of the 22 patients with recurrent glioblastoma multiforme.

Characteristic	Type	No. of patients (%), *n* = 22
Gender	Male	19 (86)
	Female	3 (14)
Age	Range	23-67
	Median	47
Region	Parietal lobe	3 (14)
	Frontal lobe	
	Temporal lobe	7 (32)
	Occipital lobe	
	Other regions	
	No data	1 (4)
Ki-67	5-10%	0
	10-20%	5
	20-50%	11
	>50%	6
MGMT	Positive	11 (50)
	Negative	10 (46)
	No data	1 (4)
IDH-1	Positive	12 (55)
	Negative	9 (41)
	No data	

### PFS and OS

**Figure 2:**
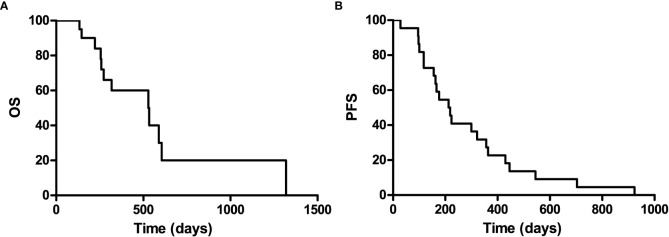
Kaplan-Meier curves for overall survival **(A)** and progression-free of the included patients **(B)**.

It is well known that patients with a recurrence GBM have a much poor prognosis, PFS and OS were calculated to indicate its prognosis. The 1-year progression-free survival was 22.7% (95% confidence interval, 13.8–31.6), and the 1-year overall survival was 60% (95% confidence interval, 48.2–71.8) for the entire study population, with a median follow-up of 529 (range 0–1,319) days (**Figure 2**).

#### Radiomic feature selection

**Figure 3:**
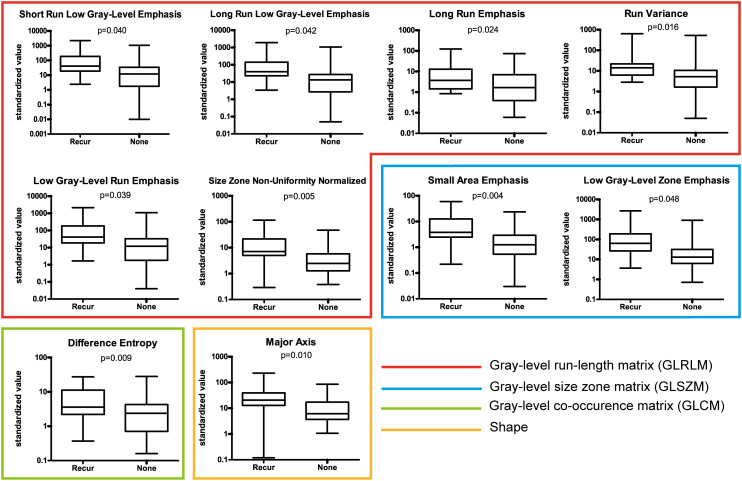
Ten features with a significant difference between the recurrence and non-recurrence regions within the peritumoral edema.

**Figure 4:**
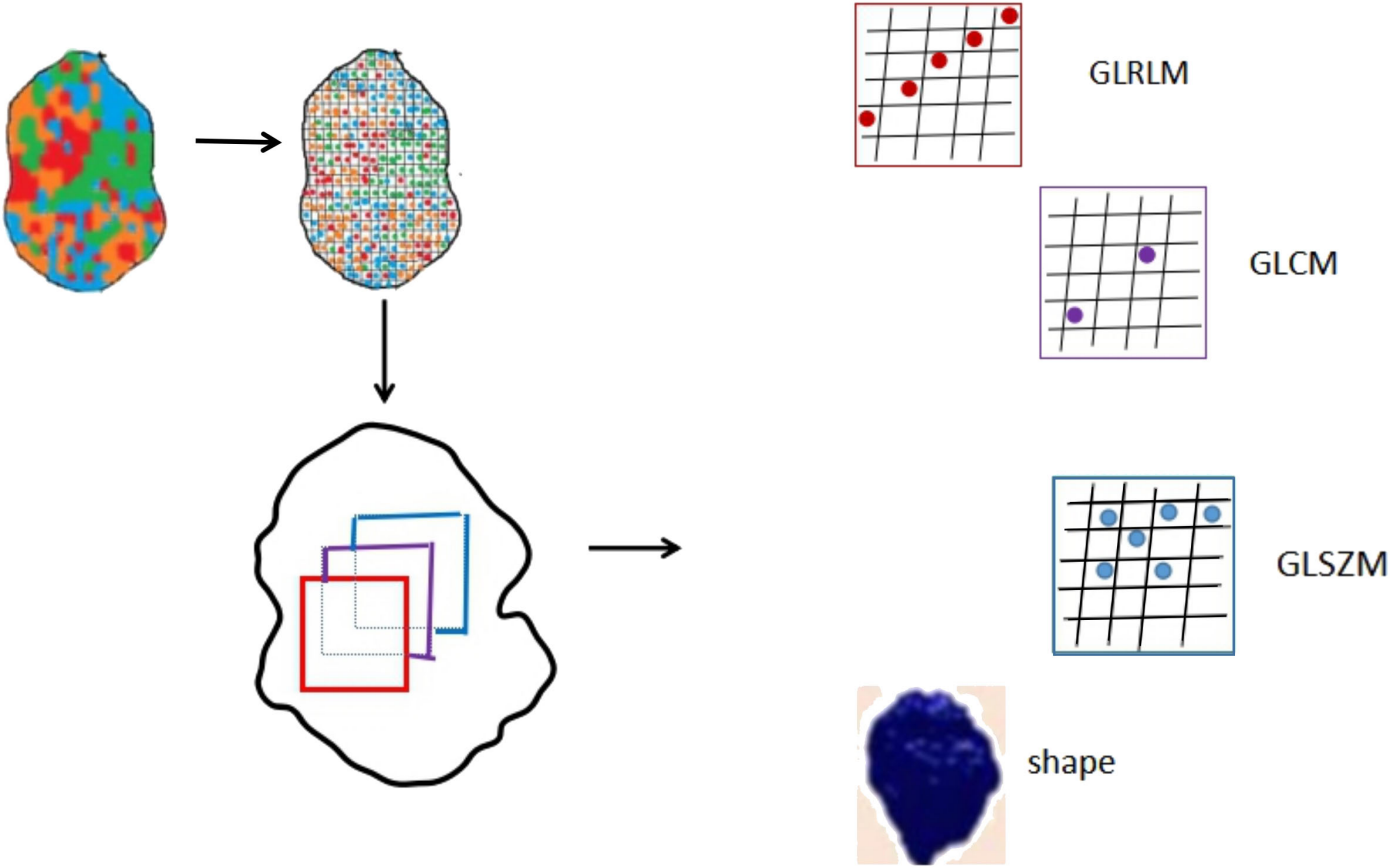
A schematic diagram presenting the findings of our study.

A set of 94 radiomic features was extracted from the recurrence and non-recurrence regions. The following 10 features demonstrated a significant difference between the recurrence and non-recurrence regions: difference entropy, short run low gray-level emphasis (15), long run low gray-level emphasis (15), run variance (16), long-run emphasis (17), low gray-level run emphasis (18), size-zone non-uniformity normalized (16), small-area emphasis (17), low gray-level zone emphasis (18),and major axis. These features belong to four different texture indexes, namely, gray-level co-occurence matrix(GLCM), gray-level run-length matrix (GLRLM), gray-level size-zone matrix (GLSZM), and shape (**Table 2**; **Figure 3**). A schematic diagram presenting the findings of our study and how the radiomic features with significant difference were calculated is shown in **Figure 4**.

**Table 2:** Radiomic features that may be relevant for predicting the site of tumor recurrence.

Texture type	Texture name	Reference(s)
GLCM	Difference Entropy	(Haralick et al 1975) (19)
GLRLM	Short Run Low Gray-Level Emphasis	(Dasarathy and Holder 1991) (15)
	Long Run Low Gray-Level Emphasis	(Dasarathy and Holder 1991) (15)
	Run Variance	(Thibault et al 2009) (16)
	Long Run Emphasis	(Galloway 1975) (17)
	Low Gray-Level Run Emphasis	(Chu et al 1990) (18)
GLSZM	Size Zone Non-Uniformity Normalized	Thibault et al 2009 (16)
	Small Area Emphasis	Galloway 1975 (17)
	Low Gray-Level Zone Emphasis	Chu et al 1990 (18)
shape	Major Axis	none

GLCM, Gray-level co-occurence matrix; GLRLM, Gray-level run-length matrix; GLSZM, Gray-level size zone matrix.

#### Verification

In order to verify the effectiveness of the 10 selected features to distinguish between recurrence and non-recurrence regions, we analyzed the FLAIR images of two patients from TCGA-GBM database with the same method as described above. Based on the hypothesis that the radiomic features of non-recurrence regions are identical to those of normal tissue outside tumor and edema, a two-sample t-test was performed to determine whether there was a significant difference in the 10 selected features between the radiomic data extracted from normal brain regions of patients in TCGA database and the non-recurrence regions of our patients. We found that seven of the 10 features showed no significant difference (*P* > 0.05),the 7 characteristics that were significantly different are difference entropy, short run low gray-level emphasis, run variance, long-run emphasis, small-area emphasis, low gray-level zone emphasis and major axis.

The acquisition of radiomic features based on GLRLM and GLSZM requires partition of the MR images (20). Some data of the recurrence regions were inevitably involved in the calculation of limbic non-recurrence regions after partition, due to the small sample size. Furthermore, the limbic voxels of the field of interest may contain normal tissue, and parts of the tumor region. For all the above reason, we believe some bias may be introduced into the calculating of radiomic features, which can be amplified by small sample patients sizes. Despite the fact that three features showed a significant difference, we still suggest that the result of our verification supports an association between the 10 identified radiomic features and the recurrence of GBM.

## Discussion

In terms of preoperative prediction for tumor recurrence, although various techniques and methods have been developed to identify different subregions (with heterogeneity) within the peritumoral edema, none of them has been routinely used with high efficacy in clinical practice (21, 22). For better prognosis of GBM, it is necessary to develop techniques of noninvasive preoperative examination for tumor recurrence. Among these, radiomics has superiority in pathological grading of gliomas and genotype definition. The molecular marker status could be reflected by imaging phenotypes, which contributed to the promotion of treatment planning before surgery and medical management (23). Similarly, radiomic features based on multi-contrast MRI can provide information correlating with the glioma grade and Ki-67 labeling index (24). It was also shown that subtypes of GBM can be identified by anatomic MRI with the biomarker volume ratio (25). Since maximum safe resection is the normal surgical treatment, a more accurate definition of the tumor volume and regions in high risk of infiltration can be developed on the base of spectroscopic MRI biomarkers, which associated radiomics with GBM treatment (26). In addition, quantitative analysis of features from MR images could reveal predictive imaging biomarkers *via* intratumoral segmentation and comparison of multimodality MR images (27). Thus, radiomics has been proven to be a bridge between imaging features and clinical treatment.

Accordingly (28), the radiomics features mainly belong to four subgroups, shape, GLCM, GLRLM and GLSZM. The GLCM depicts the spatial relationship between pixels or voxel pairs with predefined intensities of gray-level in different directions (2D analysis is in horizontal, vertical or diagonal direction, 3D analysis is in 13 directions), and distance between pixels or voxels is predefined. The GLRLM captures information of the 2- or 3-dimensional spatial distribution of continuous pixels with the same gray-level in one or more directions. Based on a calculation principle similar to GLRLM, the GLSZM captures information about spatial distribution of interconnected neighboring pixels or voxels with the same gray-level in one or more directions, in 2 or 3 dimensions. In our study, a set of 94 radiomic features was extracted from the recurrence and non-recurrence regions. Radiomics has also been applied in many studies as a preoperative noninvasive method to confirm the relationship between peritumoral edema and the prognosis of glioma (29, 30). As conventional MRI has some limitations in detection, Lemercier et al. verified the effectiveness of the apparent diffusion coefficient value gradient in differentiating glioma from metastatic lesions by means of diffusion-weighted MRI (30).

In this context, we used radiomics to investigate the heterogeneity of the peritumoral edema, considering that gliomas potentially recur in this area. Prasanna et al. extracted features from multiparametric MR images of GBM and found 10 radioactive subsets of “peritumoral” MR features that suggested intensity heterogeneity and texture patterns in the peritumoral brain region (31). In the present study, we linked radiomics with predicting the potential sites of tumor recurrence within the peritumoral edema and successfully verified that 7 radiomic features of non-recurrence regions and normal brain tissue share a relatively high similarity. Our study exhibited the potential of applying radiomics to the optimization of surgical resection for GBM.

In this study, we investigated whether there was heterogeneity within the peritumoral edema region and whether using radiomic features that display heterogeneity could roughly predict the site of GBM recurrence. Our results suggested that heterogeneity does exist in peritumoral edema, indicating that radiomic features of the peritumoral edema from routine MRI can be utilized to predict sites of GBM recurrence.

We first extracted 94 features from the peritumoral edema regions on radiological images, and through comparison between the recurrence and non-recurrence regions in the peritumoral edema, we selected 10 features exhibiting significant differences, suggesting that the peritumoral edema has certain heterogeneity. This finding is consistent with those of previous studies. At histological and cellular level, through computer-guided stereotaxic biopsies, Clavreul et al. isolated a new type of stromal cells from the peritumoral zone, named glioblastoma-associated stromal cells (1, 2). Another cell type presenting specifically in the periphery of GBM are reactive astrocytes, which are located around the tumor and may exert the function of promoting tumor growth and division (32, 33). A significant difference between the peritumoral tissue and normal brain tissue also exists at molecular level. Piwecka et al. used microarrays and sequencing to investigate the micro-ribonucleic acid (miRNA) profile in glioma tissue and peritumoral brain regions, and compare it with that of normal brain tissue. The results showed differences in the expression of 97 miRNAs between the glioma tissue and normal brain tissue (34). Similarly, Mangiola et al. used an array to detect genomic alterations and found that, compared with normal brain tissue, 15 genes were over-expressed, while 42 genes were down-regulated (35). These results suggest that although they are not as obvious as those between tumors and normal brain tissue, the differences between the peritumoral tissue and normal brain tissue cannot be ignored as well.

We also verified our results using images of two patients from TCGA database, the result of our verification supports an association between the 10 identified radiomic features and the recurrence of GBM. The purpose of our study was to find radiomics characteristics with significant difference for further prediction model establishment. Previous studies have also suggested that peritumoral edema is associated with tumor recurrence (1, 2). The downregulation of tumor suppressor genes in the peritumoral edema also suggested a correlation between peritumoral edema and tumor recurrence (35). Basen on these radiomic features acquired in this study, in another research conducted by our team, we successfully identified that satellite lesions within the peritumoral edema region of dysembryoplastic neuroepithelial tumor patients may elicit epilepsy recurrence, and radiomics could be used for detecting and evaluating these epilepsy-associated lesion (36). The differences between the peritumoral edema and normal brain tissue and those within the peritumoral edema suggest that the identification of specific regions in the peritumoral edema with relapse-related biomarkers is the basis for the prediction of tumor recurrence.

## Limitations

The major limitations of this study were the limited sample size and the single-institution design, which may reduce the efficacy of the statistical results. As a malignant central nervous system tumor, patients with GBM have a poor prognosis and low PFS and OS. Thus, the total number of patients meet our main selection criteria: pathologically confirmed diagnosis of GBM and pathologically and image confirmed tumor recurrence meanwhile accepted secondary surgey is limited. With regard to the verification, in TCGA database, the patients meet our main selection criteria mentioned above is limited and we only found two subjects available for further validation. In addition, all ROIs were conducted by one experienced neurosurgeon in this study, however, interobserver variability should be taken into account. Therefore, multicenter studies with a larger, double-blind cohort meet our selection criteria standards are required to further verify and complement our results. In addition, this was a retrospective study; thus, further prospective studies are needed to validate the clinical value of our prediction model for GBM management. In the new classification, all glioblastomas are wild-type, so there is no such entity as IDH mutant positive for glioblastoma. These are reclassified as Astrocytomas, WHO III or IV, based on histology, the patients with GBM who were diagnosis as IDH mutant type may need to be separately analyzed further.

## Conclusions

In summary, radiomics is a valuable method to determine the recurrence-related heterogeneity of peritumoral edema in order to better predict the site of tumor recurrence and help to determine the surgical resection range more accurately. However, further prospective multicenter cohort studies are required to evaluate the sensitivity and specificity of the radiomic features we have identified, which may be helpful to further guide surgical treatment to improve the prognosis of GBM.

## Data availability statement

The raw data supporting the conclusions of this article will be made available by the authors, without undue reservation.

## Ethics statement

The studies involving human participants were reviewed and approved by Ethics approval were obtained from the Ethics Committee of Nanfang Hospital Southern Mecial University. All the experiment protocol for involving human data was in accordance with the guidelines of Declaration of Helsinki. And the written consent was obtained from study participants. The patients/participants provided their written informed consent to participate in this study.

## Author contributions

HL: Research project execution, statistical analysis execution. PZ: Research project organization, statistical analysis design, manuscript review and critique. YB Statistical analysis design & execution, manuscript writing of the first draft & review and critique. CY: Statistical analysis design & review and critique. MW: Research project execution, Statistical analysis execution. DH: Research project execution, Statistical analysis review and critique. SH: Statistical analysis review and critique. KY: Research project organization, manuscript review and critique. SQ: Research project organization, manuscript review and critique. JW: Research project conception & organization, statistical analysis review and critique, manuscript review and critique. All authors contributed to the article and approved the submitted version.
